# Expression of subunits of an insecticide target receptor varies across tissues, life stages, castes, and species of social bees

**DOI:** 10.1111/mec.16811

**Published:** 2023-01-17

**Authors:** Alicja Witwicka, Federico López‐Osorio, Valentine Patterson, Yannick Wurm

**Affiliations:** ^1^ Biology Department Queen Mary University of London London UK; ^2^ Digital Environment Research Institute Queen Mary University of London London UK; ^3^ Alan Turing Institute London UK

**Keywords:** *Apis mellifera*, *Bombus terrestris*, bees, gene expression, neonicotinoid pesticides, nicotinic acetylcholine receptors, pollinator health

## Abstract

Global losses of insects jeopardize ecosystem stability and crop pollination. Robust evidence indicates that insecticides have contributed to these losses. Notably, insecticides targeting nicotinic acetylcholine receptors (nAChRs) have neurotoxic effects on beneficial insects. Because each nAChR consists of five subunits, the alternative arrangements of subunits could create a multitude of receptors differing in structure and function. Therefore, understanding whether the use of subunits varies is essential for evaluating and predicting the effects of insecticides targeting such receptors. To better understand how the use and composition of nAChRs differ within and between insect pollinators, we analysed RNA‐seq gene expression data from tissues and castes of *Apis mellifera* honey bees and life stages and castes of the *Bombus terrestris* bumble bees. We reveal that all analysed tissues express nAChRs and that relative expression levels of nAChR subunits vary widely across almost all comparisons. Our work thus shows fine‐tuned spatial and temporal expression of nAChRs. Given that coexpression of subunits underpins the compositional diversity of functional receptors and that the affinities of insecticides depend on nAChR composition, our findings provide a likely mechanism for the various damaging effects of nAChR‐targeting insecticides on insects. Furthermore, our results indicate that the appraisal of insecticide risks should carefully consider variation in molecular targets.

## 1 INTRODUCTION

Global losses of pollinating insects threaten crop yields and ecosystem stability (Goulson et al., 2015; Potts et al., 2016). Previous studies have linked these declines to insecticide exposure (Goulson, 2019; Wood & Goulson, 2017). Indeed, the predominantly used insecticides, known as neonicotinoids (Simon‐Delso et al., 2015), impair learning, immunity, foraging, and survival in bees and other pollinators (Blacquière et al., 2012; Godfray et al., 2015; Goulson et al., 2015). Although recent policies have restricted neonicotinoid use in some countries, these insecticides continue to dominate marketplaces worldwide (Klingelhöfer et al., 2022). Moreover, new, globally‐available insecticides that also target nAChRs likewise cause harm to insect pollinators (Siviter & Muth, 2020).

Neonicotinoids and related insecticides such as sulfoxaflor, flupyradifurone, spinosad, and triflumezopyrim bind to nicotinic acetylcholine receptors (nAChRs), warping the chemical messaging of insect neurons (Matsuda et al., 2020; Siviter & Muth, 2020). These receptors consist of five subunits, and an insect genome typically encodes 10‐15 nAChR subunits, which assemble into various homo‐ and heteropentameric structures (Jones & Sattelle, 2010). Consequently, every species could harbor a broad diversity of nAChRs that differ in form and function (Barrera & Edwardson, 2008; Matta et al., 2021). This variability affects toxicities because nAChRs with alternative subunit compositions differ in their affinity for insecticides (Casida, 2018; Ihara et al., 2020; Matsuda et al., 2020). For example, a mutation of the α6 subunit in the diamondback moth *Plutella xylostella* leads to mis‐splicing and production of a truncated protein, ultimately providing resistance to spinosad (Baxter et al., 2010). Similarly, a point mutation in the β1 subunit confers resistance to imidacloprid and sulfoxaflor in the peach potato aphid *Myzus persicae* (Bass et al., 2014). Furthermore, gene editing of particular nAChR subunit genes in *Drosophila melanogaster* flies changes their susceptibility to nAChR‐targeting insecticides (Lu et al., 2022; Perry et al., 2021).

Risk assessments of insecticides typically involve toxicity experiments on adult individuals of one or few surrogate species (Decourtye et al., 2013; European Food Safety Authority (EFSA), 2013; Franklin & Raine, 2019). Based on the results of such experiments, policymakers implement usage directives to safeguard thousands of beneficial insect species potentially exposed to insecticides. This extrapolation, however, largely ignores the potential diversity of molecular targets of insecticides. Uncovering substantial differences in expression of nAChR subunits within or across species would offer a plausible mechanism for the variety of damaging effects of insecticides on beneficial insects and improve our ability to assess insecticide risks.

Heritable diseases often manifest in a highly tissue‐specific manner due to multiple mechanisms, including preferential expression and post‐transcriptional processes in the susceptible tissue (Hekselman & Yeger‐Lotem, 2020). In medical research, the discovery of nAChR subtypes and control mechanisms in specific tissues has enabled the development of drugs to treat nervous system disorders (Matta et al., 2021; Taly et al., 2009). However, in the study of pollinator health, our limited knowledge of the expression of insect nAChR subunits hinders our ability to link insecticide effects to tissue‐specific receptors.

To understand how the use of nAChRs varies in pollinating insects, here we evaluate differences in expression of the nAChR gene family in two prominent species, the honey bee *Apis mellifera* and the bumble bee *Bombus terrestris*. We compare RNA‐seq gene expression data across tissues, life stages, castes, and species. Our findings indicate expression in all examined tissues, showing that nAChRs occur in both central and peripheral nervous systems. Furthermore, we reveal that expression levels of nAChR subunits and thus the potential use and composition of nAChRs differ across tissues, life stages, castes, and species. These insights indicate the fine‐tuning of nAChR expression profiles over evolutionary time. Furthermore, these results suggest that the effects of insecticide exposure on adults or developmental stages of an insect species must not be based solely on tests on adult surrogate species.

## 2 MATERIALS AND METHODS

### 2.1 Gene expression data from honey bees and bumble bees

**FIGURE 1:**
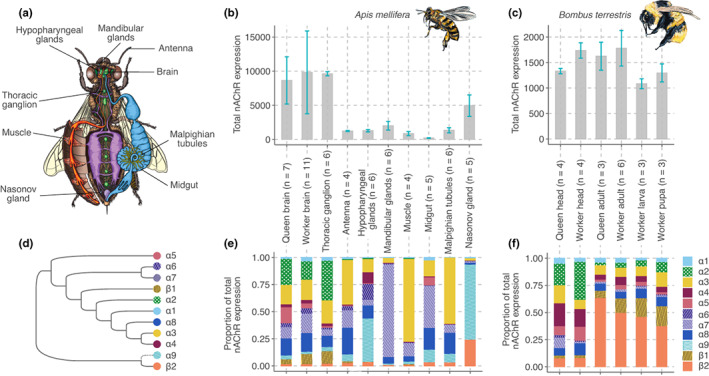
The expression of nAChR subunits differs between tissues and between castes. (a) Honey bee tissues we analysed in gene expression comparisons of nAChR subunits. (b–c) Total combined expression of nAChR subunits in honey bee (*Apis mellifera*) queen brain and nine tissues of workers (b), bumble bee (*Bombus terrestris*) queen and worker heads, and whole bodies of worker adults, larvae, and pupae (c). The bar charts show the mean of the counts and the standard deviation. Values in b and c were normalized across samples and studies using DESeq2. (d) Topological representation of the phylogeny of nAChR subunits (Jones et al., 2006); dashed line to subunit α9 indicates that the bumble bee lacks this subunit. (e–f) The ratio of expression of nAChR subunits varies in honey bees (e; 11 genes) and bumble bees (f; 10 genes) (Wald tests, FDR < 0.05). The parentheses show the number of biological replicates for each sample type.

**FIGURE 2:**
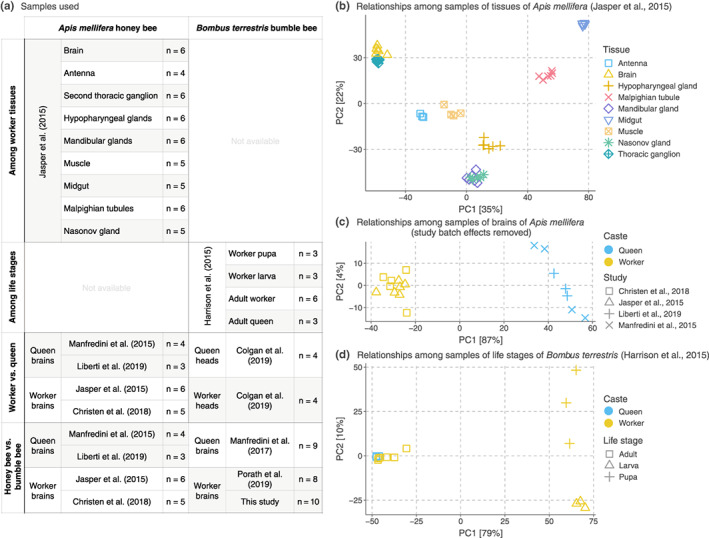
Source of and relationships among samples. (a) Datasets we analyzed in each comparison. We detected differences in the baseline levels of nAChR subunit expression using samples of untreated bees. In analyses combining data from independent studies, we used the RUVSeq method to correct potential batch effects caused due to technical differences (see Materials and Methods). (b–d) Principal component analyses show relationships among samples of (b) nine tissues of *A. mellifera* workers, (c) brains of queens and workers of *A. mellifera* corrected for the study batch effect, and (d) developmental stages of *B. terrestris*.

We retrieved RNA‐seq data from the European Nucleotide Archive. We selected data from untreated colonies (Table S1) to quantify baseline differences in gene expression. Because integrating data from multiple sources increases variability due to technical factors and the state of the colonies (e.g., age, presence of pathogens, miticide treatments, or pesticide residues), we applied measures to remove low‐quality samples and account for hidden batch effects. For *A. mellifera*, we obtained samples from nine tissues of adult workers (PRJNA243651, PRJNA211831; Jasper et al., 2015), and from brains of adult queens (Figures 1a and 2a, PRJNA275154, PRJNA52431; Liberti et al., 2019; Manfredini et al., 2015) and workers (PRJNA450296; Christen et al., 2018; Jasper et al., 2015). For *B. terrestris*, we obtained samples from heads (PRJNA508397) and brains (PRJNA497863, PRJNA358479) of adult queens and workers (Figures 1b and 2a, Colgan et al., 2019; Manfredini et al., 2017; Porath et al., 2019), and whole bodies of worker larvae, pupae, and adults, and queen adults (PRJEB9366; Harrison et al., 2015).

Furthermore, we generated primary RNA‐seq data from brains of *B. terrestris* workers (PRJNA874704). We reared workers in microcolonies from 10 queenright colonies, feeding them organic pollen and sugar water *ad libitum* every other day. We sampled 12‐day‐old workers from 10 individual microcolonies (Table S1), dissected brains on dry ice, and extracted RNA (Genaxxon Total RNA Purification Kit) from pools of three individual brains. We prepared libraries (*n* = 10, NEBNext Ultra RNA Library Prep Kit for Illumina) and sequenced them on an Illumina NextSeq 500 system, generating 40 bp paired‐end reads.

Altogether, our analyses included 20 sample types, with three to eleven biological replicates per sample type and on average 30.4 million independent RNA‐seq reads per sample. Each biological replicate was sampled from an independent colony and consisted of an untreated individual or pooled individuals. All colonies were reared following standard protocols under conditions common within each source study (Table S1).

For quality control and gene expression analyses we used Ensembl Metazoa (release 45) (Howe et al., 2020) genomes and transcriptomes of *A. mellifera* (Elsik et al., 2014) and *B. terrestris* (Sadd et al., 2015). To assess the quality of the raw RNA‐seq reads, we processed all samples using FastQC version 0.11.9 (Andrews, 2019). To estimate alignment qualities, we aligned RNA‐seq samples to the genome of the corresponding species using STAR version 2.7 (Dobin et al., 2013) and processed such alignments using the RNA‐seq module of Qualimap version 2.2.1 (Okonechnikov et al., 2016). We examined the Qualimap results to identify potential outliers based on metrics such as total number of mapped reads, transcript coverage profiles, and genomic origin of reads. To quantify gene expression, we pseudo‐aligned reads to the corresponding transcriptome using kallisto version 0.46 (Bray et al., 2016). MultiQC version 1.9 (Ewels et al., 2016) generated summaries comprising the results from FastQC, STAR, Qualimap, and kallisto. To reduce the risk of outlier samples potentially introducing biases into our analyses (Conesa et al., 2016), we excluded samples with fewer than 60% STAR‐aligned reads (i.e., outside two standard deviations of the mean; Table S1 indicates which samples we retained). For each data set, we transformed counts using the variance stabilizing transformation (VST) implemented in DESeq2 version 1.26 (Love et al., 2014) and performed principal component analysis with the top 500 most variable genes to assess the relationships of the samples (Figure 2b,c).

### 2.2 Differential gene expression

Tximport version 1.14 (Soneson et al., 2016) summarized kallisto transcript abundances to the gene‐level (Tables S4 and S5), using Ensembl (release 45) transcript‐to‐gene maps retrieved with the R package biomaRt version 2.42.1 (Durinck et al., 2009). We analysed samples of honey bee tissues, bumble bee life stages, and castes of both species separately to avoid the confounding effect caused by the source study (Figure 2a). To identify differentially expressed nAChR subunits, we applied the likelihood ratio tests (LRT) and Wald tests, as implemented in DESeq2. The LRT allows testing three or more levels of a factor at once, whereas the Wald test compares pairs of groups (the levels of a factor) and uses the estimated standard error of a log_2_ fold change to test if this expression ratio is equal to zero. We report genes as differentially expressed using a significance cutoff of 0.05 after false discovery rate adjustment (Benjamini & Hochberg, 1995) of the Wald test *p*‐values (hereafter referred to as FDR). The data of honey bee tissues and bumble bee life stages and castes originated from single source studies, rendering it unnecessary to include batch variables related to technical or experimental differences. In the comparison between the brains of honey bee castes, we used RUVSeq version 1.20 (Risso et al., 2014) to identify hidden batch effects among data from independent source studies. These effects may result from differences in library preparation, sequencing, rearing conditions, and the general state of the source colonies. We incorporated two numeric batch variables into linear models for identifying differentially expressed genes. Moreover, we applied the limma (Ritchie et al., 2015) function “removeBatchEffect()” with its “covariates” argument to remove the effects of the hidden batch variables and used counts with batch effects removed for principal components analysis (PCA; Figure 2c). Including batch factors in the models and removing their effects for PCA allowed us to investigate the effects of biological variables even when analysing data from multiple source studies.

To identify differences in the ratios of nAChR expressed subunits in an unbiased manner, for each sample we extracted DESeq2‐normalized counts for all subunits and divided them by the sum of all subunit counts. We fitted generalized linear models (logit() transformation and quasibinomial distribution) using expression proportions of nAChR subunits as the response variable and subunit, caste, and tissue as predictors.

For the comparisons between species, we normalized counts using DESeq2's median of ratios method applied to one‐to‐one orthologs of both species, accounting for library depth and composition between samples. We calculated median proportion values to perform correlation tests between the brains of queens and workers with the R function cor.test(). To check whether the source study had a significant impact, we conducted correlation tests between all individual studies.

We conducted all statistical analyses in R version 3.6.1 (R Core Team, 2019). All code underpinning these analyses is available upon publication on GitHub at https://github.com/wurmlab/2022‐amel_bter_expression_nachrs. Scripts and intermediary data will also be available at https://wurmlab.com/data/2022‐nAChR‐expression‐variation.

## 3 RESULTS

### 3.1 Bees express distinct nAChR subunits across tissues and developmental stages

**FIGURE 3:**
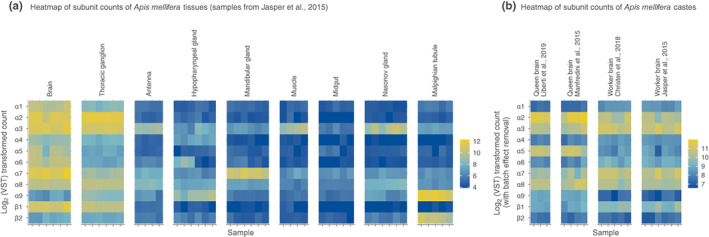
The expression levels (VST counts) of nAChR subunits in all *Apis mellifera* samples. (a) Count data of samples included in the analysis of honey bee tissues. (b) Count data of samples used in the analysis comparing brains of queens and workers, gathered from multiple sources. Removing batch effects from the transformed counts allows comparing the amounts of nAChRs subunits between castes and across source studies.

To test whether the use of nAChRs differs between tissues in honey bees, we used gene expression data from the brain, second thoracic ganglion, antennae, hypopharyngeal glands, mandibular glands, muscle, midgut, Malpighian tubules, and Nasonov gland of honey bee workers (Figure 1a). All nine tissues expressed nAChR subunits (Figures 1b and 3a), suggesting that nAChRs play key roles in the central and peripheral nervous systems of insects. However, the expression of nAChR subunits differed strikingly between tissues (Figures 1e and 3a; Table S6; likelihood ratio test, FDR < 0.05 for all subunits). For example, 64% of nAChR expression in Malpighian tubules, an important detoxification system, was from subunit α3. In contrast, 85% of nAChR expression in the mandibular glands was from subunit α7. In the Nasonov gland, 93% of nAChR expression was from subunits α9 and β2, which have divergent amino acid sequences in comparison to all other known nAChR subunits (Jones et al., 2006; Figure 1d).

To investigate the presence of nAChRs across life stages, we used gene expression data from larval, pupal, and adult bumble bee workers (Figure 2a,d). All life stages expressed nAChR subunits (Figure 1c). The expression of nAChR subunits differed substantially between life stages (Figures 1f and 2d; Table S7; likelihood ratio test, FDR < 0.05 for all subunits except α6 and α8). Therefore, toxicity estimates of nAChR‐targeting pesticides for adults probably have limited predictive power for other developmental stages.

### 3.2 Morphological and behavioral castes differ in the expression of nAChRs

Superorganismal colonies of social bees, such as bumble bees and honey bees, include morphologically distinct queen and worker castes (Danforth et al., 2013). To test the hypothesis that castes differ in their use of nAChRs, we compared the expression of nAChR subunits between the brains of honey bee queens and workers and between the heads of bumble bee queens and workers. A principal components analysis using batch‐corrected counts of honey bees revealed caste as the largest source of variance (Figure 2c). In both species, queens and workers differed in their use of nAChR subunits (Figures 1e,f and 3b; Wald tests, FDR < 0.05, Tables S2 and S3).

We also compared nAChR expression between honey bee nurses and foragers, two types of workers that differ only in behavior. We found differences in the hypopharyngeal glands (α3 and α6), mandibular glands (α8 and α6), midgut (α5), and Malpighian tubules (α8 and α9) (Wald tests, FDR < 0.05, Table S2).

The differences in nAChR subunit use between colony members help explain why nAChR‐targeting insecticides have caste‐specific effects (Colgan et al., 2019), suggesting that we cannot extrapolate the results of toxicity tests between castes.

### 3.3 Expression of nAChR subunits differs between bee species

**FIGURE 4:**
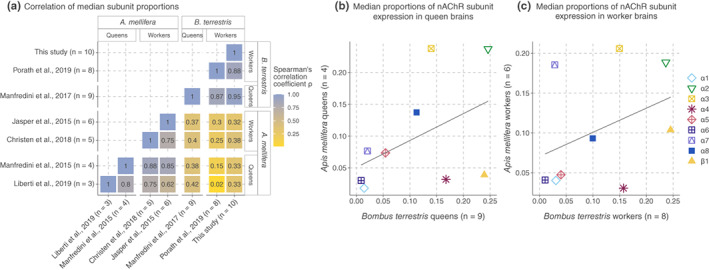
Differences in nAChR subunit expression between species. Interspecies comparisons excluded the divergent subunits α9 and β2 and relied solely on the single‐copy orthologous subunits. Pairwise comparisons of median subunit proportions revealed high positive correlations (Spearman's coefficients) between independent studies of each caste per species; numbers indicate Spearman's ρ values (a). The use of nAChR subunits differs between honey bees and bumble bees in the brains of queens (b) (Spearman's ρ = 0.52, *p* = 0.16) and workers (c) (Spearman's ρ = 0.30, *p* = 0.44); we fitted the regression lines using the least squares method.

To understand how much nAChR expression differs between species, we compared the expression of nine nAChR subunit orthologs between the brains of honey bees and bumble bees, excluding the divergent subunits α9 and β2. Pairwise comparisons of median ratios of subunit expression counts revealed major differences between species (Figure 4). Subunit expression was uncorrelated between queens (Spearman's ρ = 0.52, *p* = 0.16; Figure 4b) and between workers (Spearman's ρ = 0.30, *p* = 0.44; Figure 4c). Because study‐specific technical factors can confound comparisons between species, we also compared independent studies of the same caste and tissue. Unlike the between‐species comparisons, expression ratios correlated highly between separate studies within each species (Figure 4a). The differences between species draw into question the current pesticide testing practices, which rely heavily on only one or a few species as representatives of all pollinators (European Food Safety Authority [EFSA], 2013).

## 4 DISCUSSION

This study reveals spatial and temporal patterns in the transcriptional control of nAChR subunits in social bees. Our findings should help identify the mechanistic bases of the damaging effects of insecticides on insect pollinators. We detected the expression of nAChRs in all studied tissues, indicating that these receptors play critical roles in the central and peripheral nervous systems of insects (Thany et al., 2010). The widespread expression of nAChRs can explain why nAChR‐targeting insecticides disrupt broad aspects of biology, including immune functions, reproductive output, learning abilities, motor control, and sensory capabilities (Blacquière et al., 2012; Godfray et al., 2015; Goulson et al., 2015; Wood & Goulson, 2017). Furthermore, we found substantial differences in the relative abundances of nAChR subunits across tissues, life stages, castes, and species. These differences suggest that natural selection has fine‐tuned receptor composition in tissue‐, caste‐, and species‐specific manners over evolutionary time.

Differences in amino acid sequences between nAChR subunits determine their biochemical properties and three‐dimensional structures (Stokes et al., 2015). In turn, these differences govern the physiological and pharmacological properties of nAChRs (Casida, 2018; Jeschke et al., 2011), including their binding affinities for the essential neurotransmitter acetylcholine and for so‐called cholinergic insecticides including neonicotinoids and some of their alternatives (Casida, 2018; Ihara et al., 2020; Matsuda et al., 2020; Taillebois et al., 2018). For example, mutations that lead to changes in amino‐acid sequence, splicing, or presence of particular nAChRs change susceptibility to nAChR‐targeting insecticides in insects (Bass et al., 2014; Baxter et al., 2010; Perry et al., 2021). Similarly, due to structural differences between insect and mammalian nAChRs, the latter have lower affinities for neonicotinoids (Tomizawa & Casida, 2005).

A combination of neutral processes, functional constraints, and adaptation probably drive the specialized use of alternative nAChR subunit assemblies across tissues, castes, and species. Results from studies of mammals reveal that nAChR subunits in different cell types underwent alternative evolutionary trajectories characterized by sequence divergence and differences in co‐expression patterns (Marcovich et al., 2020). Likewise, human studies show non‐neuronal, tissue‐specific expression patterns of nAChRs (Zhang et al., 2017). In insects, natural selection could favor changes in nAChR subunit composition to reduce the impact of nAChRs binding natural plant compounds from nectar or pollen or to improve physiological functions.

Although expressed genes cannot reveal the composition of functional receptors, the co‐expression of subunits limits the potential variety of receptor assemblies in any given tissue and developmental stage. Evidence from the fruit fly *Drosophila melanogaster* and the mouse *Mus musculus* indicates that patterns of co‐expressed subunits correlate positively with the coassembly of nAChR subunits (Croset et al., 2018; Saunders et al., 2018). Several other processes probably affect the structures and functions of nAChRs, including differences in alternative splicing, RNA editing, post‐translational modification, and the presence of accessory proteins enabling distinct coassemblies of subunits. The differences in such other processes could further modulate the pharmacological properties of receptors, and thus the range of insecticide exposure impacts within and across species. Further work on the interplay between transcriptional control and other molecular mechanisms would offer a complementary view of the variability in expressed nAChR subunits.

Similarly, the differences in coexpressed nAChR subunits reported here can help explain why insecticides cause a spectrum of damaging symptoms which vary across species and exposure modes (Blacquière et al., 2012; Godfray et al., 2015; Goulson et al., 2015; Wood & Goulson, 2017). The variety of patterns in nAChR expression and differences in composition highlight the importance of acetylcholine‐based signalling in bees and indicate how insecticide exposure could disrupt organismal function. For example, nAChR subunit expression in larvae (Figure 1c,f) reinforces the pivotal role of cholinergic signalling in development. This finding concurs with evidence of workers providing acetylcholine to developing larvae (Wessler et al., 2016). Independent work indicates a dynamic landscape of expressed nAChR subunits during development and damaging effects in insect larvae exposed to neonicotinoids (Pyakurel et al., 2018; Smith et al., 2020; Wu et al., 2017). Our study revealed significant differences in the expression of nAChR subunits between life stages of untreated bumble bee workers (Table S2). Similarly, in the aphid *Acyrthosiphon pisum*, transcript levels of nAChR subunits α1‐3, α6, α8‐9, and β2 fluctuated while subunits α7, α10, and β1 remained stable during larval development (Taillebois et al., 2014). The pattern that insects differentially regulate nAChR subunits throughout development could explain why bees exposed to neonicotinoids during early life stages develop smaller brains and experience impaired learning (Smith et al., 2020), immune responses (Annoscia et al., 2020; Di Prisco et al., 2013), foraging patterns, and reduced lifespan (Tsvetkov et al., 2017). In *Drosophila* larvae, exposure to the neonicotinoid imidacloprid degenerates neurons and induces vision loss (Martelli et al., 2020). These effects correlate with reports of distinct transcriptional control of subunits Dα1 and Dα6 in the visual system of *Drosophila* larvae during development and the critical roles of these two subunits in dendrite morphogenesis and transmitting synapses (Rosenthal et al., 2021).

The high expression of nAChR subunits in glands of the honey bee indicates that cholinergic signalling stimulates glands and suggests that altering such signalling may have multifaceted effects. The mandibular glands, for example, can produce alarm pheromones in foraging honey bee workers (Nouvian et al., 2016), whereas the queen mandibular gland pheromone inhibits ovary development in workers, thus contributing to regulating social organization within the colony (Conte & Hefetz, 2007; Princen et al., 2019). Moreover, the mandibular glands of honey bee foragers express high amounts of immunity genes (Vannette et al., 2015), suggesting a possible basis for the adverse effects of insecticides on the insect immune system (Annoscia et al., 2020; Brandt et al., 2016). In addition, insecticides acting in the Nasonov gland could affect the release of an essential pheromone that guides workers to their colony and food sources (Reinhard & Mandyam, 2009). Lastly, hypopharyngeal glands, with known roles in behavioral specialization, differed in nAChR expression between honey bee foragers and nurses (Table S2). It is plausible that this difference could be linked to alternate roles of hypopharyngeal glands: these glands produce nectar‐processing enzymes in foragers, while in nurses these glands synthesize royal jelly proteins for feeding larvae (Ueno et al., 2015). Such results highlight another mechanism through which nAChR‐targeting insecticides may hinder division of labor, paralleling a report that neonicotinoids reduce the expression of genes encoding major royal jelly proteins (Wu et al., 2017).

Strikingly, we found considerably high amounts of the divergent subunits α9 and β2 in the Nasonov gland relative to the other honey bee tissues. These two nAChR subunits have divergent amino acid sequences compared to all other known nAChR subunits (Jones et al., 2006) (Figure 1d), suggesting both may have evolved distinct functions in a gland unique to bees (Figure 1d,e). Likewise, subunit β2 represented a much higher proportion of nAChR expression in whole bodies of adult and immature bumble bees than in heads (Figure 1f). These results convey that the most divergent subunits may be essential for nAChR functions in novel tissues beyond the central nervous system.

Our findings have two important implications for insecticide safety practices, which rely heavily on examining adult individuals of one or a few species as representatives of all pollinators (European Food Safety Authority [EFSA], 2013). First, the differences we report among tissues and life stages suggest that standard toxicity testing on adult worker bees has limited predictive power and cannot reflect the full range of effects within a species. Second, differences in subunit expression between species emphasize the difficulty of generalizing toxicity estimates across species. This finding complements reports of variation among species in insecticide susceptibilities (Wood & Goulson, 2017) and repertoires of detoxification genes (Hayward et al., 2019; Manjon et al., 2018).

Our findings underline the promise of molecular approaches to dissect the intricacies underlying adverse insecticide effects and advancing pollinator health (López‐Osorio & Wurm, 2020). Overall, this study challenges the standard practice of applying broad‐spectrum insecticides because appropriately demonstrating the safety of such compounds demands a greater understanding of their molecular targets.

## AUTHOR CONTRIBUTIONS

Alicja Witwicka, Federico López‐Osorio, Valentine Patterson and Yannick Wurm designed the research. Alicja Witwicka, Federico López‐Osorio and Valentine Patterson performed the research. Alicja Witwicka, Federico López‐Osorio and Valentine Patterson analysed the data. Alicja Witwicka and Federico López‐Osorio visualized the data and results. Alicja Witwicka, Federico López‐Osorio and Yannick Wurm wrote the manuscript. Yannick Wurm supervised the research. Alicja Witwicka, Federico López‐Osorio, Valentine Patterson and Yannick Wurm approved the manuscript.

## CONFLICT OF INTEREST

The authors declare no conflict of interest.

## Supporting information


**Table S1.** Overview of all analyzed samples.

**Table S2.** DESeq2 Wald test results for *Apis mellifera*. The column "log2FoldChange" is the effect size estimate. This value describes how much the gene’s expression changed between groups. This value is reported on a logarithmic scale to base 2 (a log_2_ fold change of 1 means a two‐fold increase in gene expression). The first term in the "Comparison" column is the group in the numerator. The column "AdjustedPvalue" is the *p* value adjusted for multiple testing using the procedure of Benjamini and Hochberg (1995).

**Table S3.** DESeq2 Wald test results for *Bombus terrestris*. The column "log2FoldChange" is the effect size estimate. This value describes how much the gene’s expression changed between groups. This value is reported on a logarithmic scale to base 2 (a log_2_ fold change of 1 means a two‐fold increase in gene expression). The first term in the "Comparison" column is the group in the numerator. The column "AdjustedPvalue" is the *p* value adjusted for multiple testing using the procedure of Benjamini and Hochberg (1995).

**Table S4.** *Apis mellifera* transcript abundances summarized to the gene‐level.

**Table S5.** *Bombus terrestris* transcript abundances summarized to the gene‐level.

**Table S6.** DESeq2 Likelihood ratio test results for *Apis mellifera*. The likelihood ratio test (LRT) allows testing three or more levels of a factor at once. The column "AdjustedPvalue" is the *p* value adjusted for multiple testing using the procedure of Benjamini and Hochberg (1995).

**Table S7.** DESeq2 Likelihood ratio test results for *Bombus terrestris*. The likelihood ratio test (LRT) allows testing three or more levels of a factor at once. The column “AdjustedPvalue” is the *p* value adjusted for multiple testing using the procedure of Benjamini and Hochberg (1995).

## Data Availability

Sequence data underlying this work have been made available in the European Nucleotide Archive and NCBI Short Read Archive (accessions PRJNA874704, PRJNA243651, PRJNA211831, PRJNA275154, PRJNA524311, PRJNA450296, PRJNA508397, PRJNA497863, PRJNA358479, PRJEB9366). Analysis scripts have been made available on GitHub at https://github.com/wurmlab/2020‐amel_bter_expression_nachrs. Data and scripts are also available at https://wurmlab.com/data.
